# Platelet-Cancer Interplay: Molecular Mechanisms and New Therapeutic Avenues

**DOI:** 10.3389/fonc.2021.665534

**Published:** 2021-07-12

**Authors:** Attila Braun, Hans-Joachim Anders, Thomas Gudermann, Elmina Mammadova-Bach

**Affiliations:** ^1^ Walther-Straub-Institute for Pharmacology and Toxicology, Ludwig-Maximilian-University, Member of the German Center for Lung Research (DZL), Munich, Germany; ^2^ Division of Nephrology, Department of Medicine IV, Ludwig-Maximilians-University Hospital, Munich, Germany

**Keywords:** platelets, cancer, thrombosis, anti-platelet therapies, metastasis

## Abstract

Although platelets are critically involved in thrombosis and hemostasis, experimental and clinical evidence indicate that platelets promote tumor progression and metastasis through a wide range of physical and functional interactions between platelets and cancer cells. Thrombotic and thromboembolic events are frequent complications in patients with solid tumors. Hence, cancer modulates platelet function by directly inducing platelet-tumor aggregates and triggering platelet granule release and altering platelet turnover. Also, platelets enhance tumor cell dissemination by activating endothelial cell function and recruiting immune cells to primary and metastatic tumor sites. In this review, we summarize current knowledge on the complex interactions between platelets and tumor cells and the host microenvironment. We also critically discuss the potential of anti-platelet agents for cancer prevention and treatment.

## Introduction

Cancer is caused by uncontrolled cell division and growth of malignant cells, which can spread throughout the body, resulting in metastasis (1, 2). For years, cancer biology focused on tumor cell attributes, leading to life-threatening complications. Initial studies on tumor suppressor genes and oncogenes fostered our understanding of the basic mechanisms of tumorigenesis and associated cell signaling pathways that lead to tumor cell malignancy. In recent years, many studies provided evidence that tumor progression is not tumor-cell autonomous, but rather involves cellular and molecular cross-talk with the different components of the surrounding tumor environment (3, 4). This tumor microenvironment is formed of complex tissues that contain extracellular matrix (ECM), cytokines, growth factors, and adhesion molecules, also diverse cellular components such as fibroblasts, immune cells, adipocytes, pericytes, epithelial cells, lymphatic and endothelial cells and platelets (5). Stimulated crosstalk between tumor and the surrounding environment involves the recruitment of various cell types, remodeling of the ECM, as well as stimulating the immune and coagulation system (6). The tumor microenvironment provides the necessary milieu, nutrients, and blood supply which stimulates tumor cell spreading and metastasis throughout the body.

Platelets are small anucleated fragments derived from megakaryocytes in bone marrow sinusoids, circulating in the blood, which play a critical role in thrombosis and hemostasis, arrest of bleeding in healthy conditions. Upon vascular injury, (i) the exposure of subendothelial matrix proteins to the blood flow, (ii) anchoring von-Willebrand-Factor (vWF) to the matrix and platelet surface, thereby (iii) inducing platelet glycoprotein (GP)Ibα–vWF interaction and subsequent (iv) GPVI–collagen interaction, are crucial steps in platelet adhesion and thrombus formation (7, 8). Platelets express several integrins on the surface, which interact with various ligands, including fibrinogen, vitronectin, collagen, fibronectin, and laminin, which mediate platelet attachment to the vessel wall. In secretory granules, platelets store several bioactive plasma proteins (coagulation factors, fibrinogen, vWF), regulatory factors and secondary mediators, such as adenosine di- and triphosphate (ADP/ATP) and serotonin, which are released upon platelet activation, thereby enhancing pro-thrombotic events, stimulating the recruitment of circulating platelets to the site of injury (8, 9). Platelet accumulation at the site of vascular injury triggers platelet aggregation and blood clotting, generating thrombin and active coagulation factors. This process is regulated by the extrinsic and intrinsic coagulation pathways. Upon the action of thrombin, soluble fibrinogen is converted to fibrin, which enhances platelet activation and aggregation responses. Activated platelets expose phosphatidylserine (PS) facilitating the recruitment of the prothrombinase complex, thereby connecting the outer platelet surface to components of the coagulation cascade (9–11).

Thrombotic events have been frequently observed in cancer patients indicating an active involvement of platelets and factors released from platelets in tumor progression, enhancing pro-coagulant activity and blood clotting (12, 13). Although the systemic effects of platelets in thrombotic complications of cancer patients have been described, compelling experimental and clinical evidence linked platelet function to tumor angiogenesis, tumor progression and metastasis through the interaction of platelets with cancer cells and tumor microenvironment. However, the direct involvement of platelets in the tumor-forming microenvironment has not yet been convincingly demonstrated (14). Several open questions have to be answered before we can begin to understand the molecular mechanisms of platelet-dependent tumor growth and metastasis. In the first part of this review, we summarize several concepts that we described in 2015 (14) by presenting new experimental and conceptual progress about the role of platelets in different steps of tumor progression, including the molecular mechanisms of cancer-associated thrombosis and thrombo-inflammation, tumor angiogenesis and metastasis. In the second part of our review, we discuss the advantage of the clinical diagnosis of platelet-related molecular and cellular signatures and highlight anti-platelet therapies to avoid hemostatic complications in cancer patients, and to increase the efficacy of anti-cancer therapies.

## Cancer-Associated Thrombosis and Thrombo-Inflammation

Cancer patients often suffer from thrombotic complications, such as deep vein or arterial thrombosis, and pulmonary emboli. Thromboembolic disease is the second leading cause of death in cancer patients diagnosed with obesity, leukocytosis, anemia or thrombocytosis. Thrombocytosis is associated with poor survival and increased risk of tumor metastasis and venous thromboembolism (VTE) in a wide variety of cancers, including colorectal, breast, lung, renal and gastric cancers (15–18). Depending on the disease state of cancer, patients have to face a 4 to 8 times greater risk of venous thrombosis or thromboembolism compared to patients without cancer (18). However, the exact molecular mechanisms of thrombocytosis and other aforementioned pathological complications are only partially understood. Approximately one-third of newly diagnosed ovarian cancer patients have exceedingly high platelet counts that are associated with shortened survival (19).

**Figure 1:**
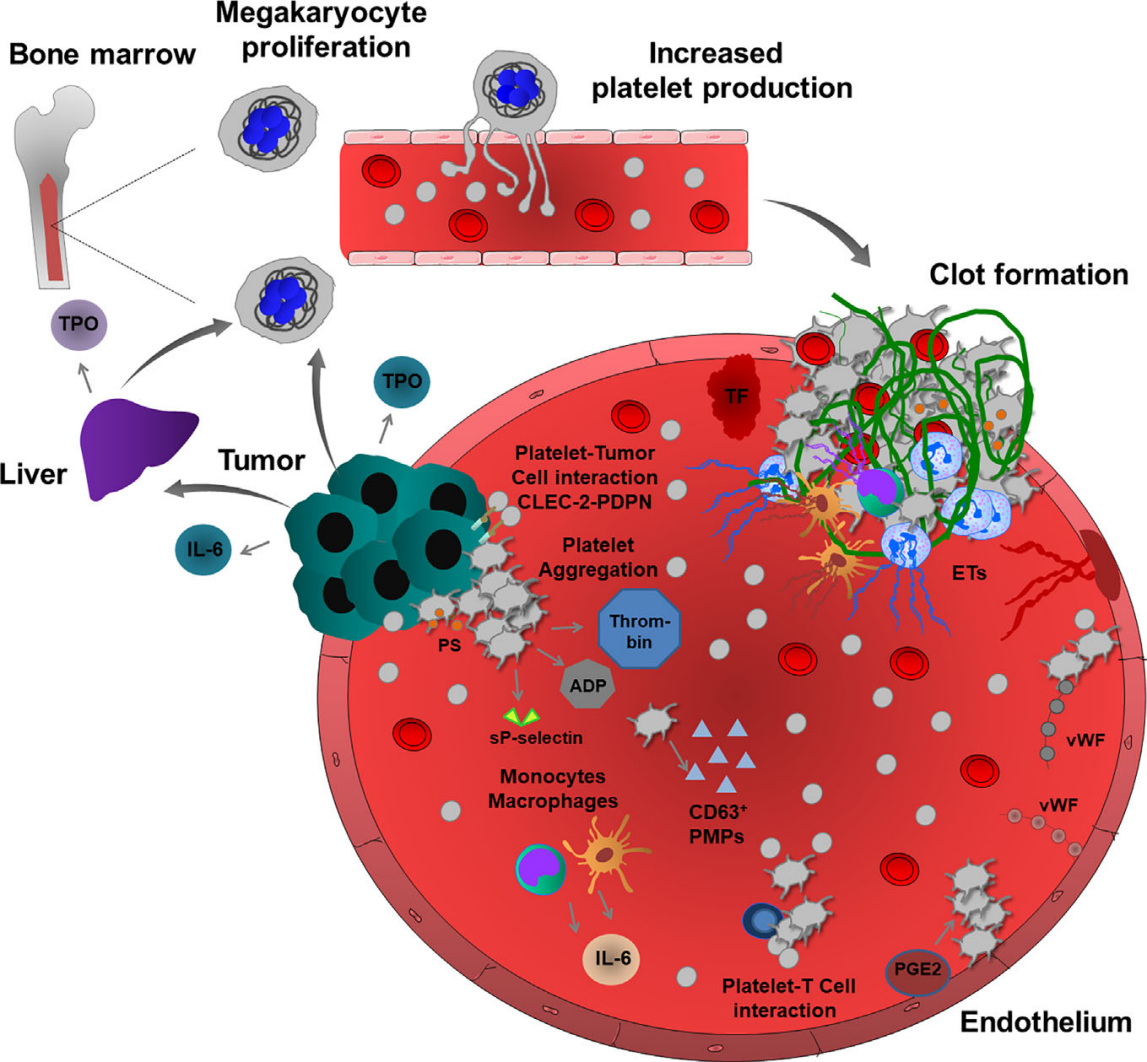
Cancer-associated thrombosis and thrombo-inflammation. Growing tumors can induce thromboembolic events through several mechanisms. Tumor and immune cells (monocytes and macrophages) release inflammatory cytokines, such as IL-6, which regulate TPO levels in the liver, thereby enhancing the proliferation of megakaryocytes and consequent platelet production. Some tumor cells can also produce TPO, thereby increasing platelet production. In a pro-thrombotic tumor environment, TF and platelet and endothelial cell-derived vWF enhance platelet activation and aggregation. CLEC-2-PDPN-mediated interactions enhance platelet-tumor cell cross-talk and TCIPA. Activated platelets release many pro-coagulant factors and also sP-selectin and ADP. Increased thrombin generation and PS – exposure on the surface of activated platelets induce intravascular clotting and thrombosis. In addition, platelets and tumor microenvironment activate immune cells, such as neutrophils, monocytes, macrophages and also endothelium thereby promoting the release of ETs, further inducing inflammation, platelet activation, aggregation and fibrin-rich clot formation. During cancer progression, platelets also interact with T cells to enhance thrombosis. In addition, endothelial PGE2, PMPs and extracellular vesicles can also enhance platelet activation and induce thrombosis and thrombo-inflammation in growing tumors.

Several studies suggested a molecular mechanism of the development of cancer-associated thrombocytosis which can be explained by the ability of some cancer cell types to produce thrombopoietin (TPO), a key cytokine stimulating megakaryocyte differentiation and proliferation and resultant platelet production. Elevated serum levels of TPO were observed in cancer patients with reactive thrombocytosis (19, 20). Interestingly, in many cases, cancer patients with high plasma levels of TPO also had increased production of interleukin (IL)-6, and both parameters were linked to advanced disease and poor survival (19, 21). Accordingly, in mouse models of colorectal and ovarian carcinoma, the inflammatory response of tumor and immune cells involves IL-6 production that can stimulate platelet production by enhancing TPO secretion from hepatocytes (19, 22). This pathological process is inhibited in IL-6-deficient mice, confirming the paraneoplastic effect of IL-6 in colorectal carcinoma-induced thrombocytosis (22). Additionally, ovarian cancer cells can secret functionally active TPO, directly affecting platelet production in the bone marrow (23). Besides thrombocytosis, cancer patients present with elevated expression of platelet-derived markers, including CD40 and β-thromboglobulin (24). P-selectin exposed on the activated platelet surface and soluble form are increased in the blood, and this increased level was associated with VTE in cancer patients (24–26). Moreover, cancer patients frequently have high levels of CD63-positive platelet-derived microparticles (PMPs), inducing a pro-coagulant cancer environment (**Figure 1**) (27, 28).

Cancer cells can directly activate platelets and enhance thrombus formation. Tumor cell-induced platelet activation and aggregation (TCIPA) has been detected *in vitro* using neuroblastoma, small-cell lung, fibroblastoma, renal, gastric, melanoma, breast and colorectal cancer cells (29). Cancer cell-resident podoplanin (PDPN) was proposed as a key regulator of this process. PDPN is a type I transmembrane sialomucin-like glycoprotein located on the surface of many tumor cells, including squamous cell carcinoma, seminoma and brain cancer cells (30). Increased expression of PDPN in cancer cells is associated with a high risk of thrombosis (30). Overexpression of PDPN in vascular endothelial cells could also induce thrombo-inflammation, possibly promoting PDPN-induced cellular interactions between platelets, cancer cells and endothelial cells. Platelet aggregation was enhanced by PDPN-positive oral squamous carcinoma cells, and mice developing PDPN-positive tumors have a reduced survival rate (31). Podoplanin expression by human brain tumors also induces platelet aggregation and is associated with hypercoagulability and a high risk of VTE (32). C-type lectin-like receptor-2 (CLEC-2) was originally discovered as an important platelet hemi-immunoreceptor tyrosine-based activation motif (ITAM) receptor, which is activated by snake venom toxin rhodocytin and PDPN. Inhibition of platelet CLEC-2 function in a mouse model of lung cancer significantly reduces thrombus formation and metastatic events after injection of B16F10 melanoma cells, suggesting that interaction between platelet CLEC-2 and cancer-resident PDPN may also enhance thromboembolism, TCIPA and platelet-dependent tumor cell spreading in human patients (33).

Cancer cells can also trigger indirect platelet activation by enhancing the release of ECM proteins and tissue factor (TF) from endothelial cells, building an active surface for platelet adhesion and thrombus formation (34). Platelet-dependent thrombin generation and consequent protease-activated receptor (PAR) activation, phospholipase C (PLC) activation, calcium store depletion, and activation of the small guanosine-5’-triphosphate (GTP)-ase Rap1b signaling have been detected in this process. Inhibition of PLC in platelets could prevent TCIPA, indicating the major route of inositol trisphosphate (IP_3_)-dependent calcium store release and diacylglycerol (DAG)-mediated signaling in this process, probably acting on DAG/protein kinase C(PKC)-mediated Rap1b activation (35). IP_3_-dependent calcium store release also induces PS exposure on the platelet surface, activating the prothrombinase complex. In line with this, PS-positive platelets were found to be significantly higher in the blood samples of cancer patients, resulting in shorter blood clotting time and increased prothrombinase activity (28, 36).

Cancer-associated thrombosis can be induced independently of TF. Gas6 is a vitamin K-dependent ligand for the receptor tyrosine kinase family comprising Tyro3, Axl and Mer (TAM), acting as pro-survival factors in both tumor and endothelial cells (37). Although Gas6 regulates inflammatory functions in immune and endothelial cells, in lung cancer cell-associated venous thrombosis model, Gas6 enhanced prostaglandin E2 (PGE2) secretion from the endothelium leading to platelet activation and venous thrombosis (37). The interactions of platelets with T cells also contribute to an inflammatory pro-coagulant phenotype and thrombosis in patients with lung cancer (38).

The inflammatory response often results in increased levels of vWF released by activated platelets and endothelial cells, e.g. in post-operative patients with malignant prostate cancer (39). The thrombogenic lesions also increase the risk of TCIPA after surgical intervention (39). Interestingly, deficiency or inhibition of androgen receptor function in prostate cancer cells could induce TCIPA *in vitro* (40). Conclusively, the loss of androgen receptors in cancer cells accounted for the increased thrombogenicity, due to the enhanced expression of prothrombin. In sharp contrast, androgen receptor-positive prostate cancer cells cannot induce TCIPA (40). Mitrugno et al. reported that FcγRIIa expressed on human platelets can mediate P3 prostate cancer cell-induced platelet activation and that these tumor cells directly induce ADP release (41). Interestingly, this platelet-tumor cell crosstalk is also induced by direct interaction of platelet FcγRIIa with cancer cell-derived immunoglobulin G (42).

Neutrophil extracellular trap (NET) formation is frequently observed in cancer patients, increasing levels of histones, deoxyribonucleic acid (DNA), and other nucleosome compounds in the blood. NET release is associated with the incidence of cancer-associated thrombosis and organ failure, mainly triggering cancer-related coagulopathy (43). NET release was proposed as a causative factor in pancreatic cancer and more than 25% of cancer patients develop VTE (44). Increased levels of TF, extracellular vesicles, citrullinated histone H3 and extracellular vesicle TF activity were observed in these patients. Using different *in vivo* experimental settings, inhibition of TF, depletion of neutrophils, or administration of deoxyribonuclease I (DNAse I) in mice could inhibit venous thrombosis (45). These results suggest that systemic DNAse I treatment degrading NETs can inhibit cancer-associated thrombosis and tumor growth. Interestingly, in many experimental models, DNAse I treatment, but not neutrophil depletion could inhibit tumor growth and thrombosis, indicating alternative sources of ETs (46–50). Increasing evidence suggests that monocytes, macrophages and endothelial cells can also extrude their granular and nuclear content and in some cases, activated platelets contribute to the process of ETosis (48, 50–52). Pro-coagulant cancer cells can also release ETs (53). Altogether, these results suggest that platelets and their granular content may contribute to the formation of ETs, supporting thrombus formation and coagulopathy in cancer patients.

## Platelets and Vascular Network of Tumors

### Vascular Sprouting

After reaching a certain size, solid tumors need to stimulate angiogenesis, receiving more nutrients and growth factors, which are required for energy metabolism, signaling and tumor growth (54). Tumor angiogenesis includes vessel sprouting, intussusceptive endothelial cell growth, remodeling and differentiation into arterioles, venules and capillaries. Vascular sprouting is a tightly regulated process involving the action of motile tip cells at the leading edge, that migrate towards pro-angiogenic signals and guided by pro-angiogenic vascular endothelial growth factor (VEGF) and proliferating stalk cells, elongate vascular sprout and generate the vessel lumen. This fully formed vessel recruits mural cells, pericytes and vascular smooth muscle cells, and promotes vessel integrity and blood perfusion (54).

**Figure 2:**
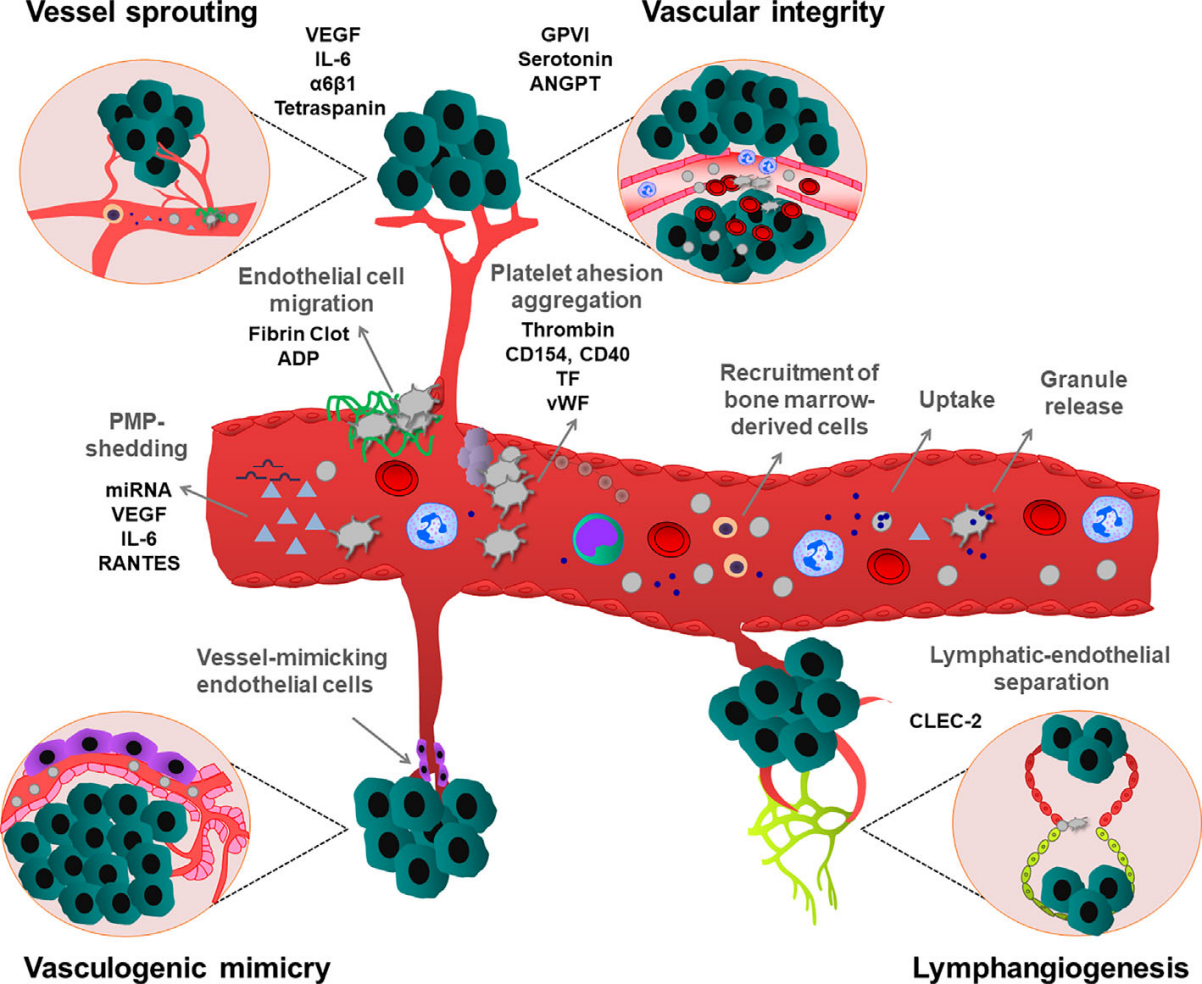
Effects of platelets on different types of tumor angiogenesis and maintenance of vascular integrity. Platelets stimulate tumor neovascularization by multiple mechanisms. Platelets induce secretion of pro-angiogenic growth factors, from tumor and tumor microenvironment. Platelets can also uptake and sequester pro-angiogenic mediators from the tumor and surrounding environment and deliver and release them into the tumor. Additionally, PMPs enhance vessel formation. Platelets also stimulate the recruitment of bone marrow-derived cells to the angiogenic tumor tissues. Platelets attach to activated endothelium, thereby inducing the migration of endothelial cells on a platelet-rich fibrin matrix. Besides, platelets can maintain the integrity of tumor vessels, preventing hemorrhages, thereby enhancing the survival of growing tumors. Platelets also maintain the lymphatic-endothelial separation during tumor lymphangiogenesis. In contrast to their activatory roles in other angiogenesis types, vasculogenic mimicry-induced tumor angiogenesis is inhibited by platelets. As shown, all these processes can be regulated by platelet-mediated processes involving different cellular and molecular mediators.

Platelet α-granules are the major store of the angiogenic factors that simultaneously control hemostasis and angiogenesis in the tumor microenvironment (55) (**Figure 2**). Activated platelets release α-granule-resident pro-angiogenic factors, such as VEGF, epidermal growth factor (EGF), basic fibroblast growth factor (bFGF) and also release anti-angiogenic factors, such as angiopoietin-1 (ANGPT1), sphingosine 1-phosphate (S1P), thrombospondin-1 (TSP1) and endostatin. Depending on external stimuli, platelets can selectively release these factors to stimulate or inhibit vessel formation in the growing tumor. For instance, ADP-stimulated platelets can release VEGF, but not endostatin, while thromboxane A_2_ (TxA_2_) stimulation induces more endostatin release than VEGF *in vitro* conditions (56). ADP-stimulated platelet-releasate promotes the capillary formation of human umbilical vein endothelial cells (HUVEC), while thromboxane A_2_ (TxA_2_)-stimulated platelet-releasate inhibits this process (56). Platelet granule release may have anti-angiogenic effects in the tumor microenvironment, because higher endostatin, TSP1, angiostatin levels were measured in serum and urine of different cancer patients (57–59). However, the concept of co-clustering of proteins in distinct granules and differential granule release was challenged by several studies using quantitative enzyme-linked immunosorbent assay (ELISA), confocal immunofluorescence microscopy and proteomic approaches (60–64), which did not observe any functional pattern. Recently, it has been proposed that Stimulated Emission Depletion (STED) imaging can be applied to study platelet granule content and protein clustering in a more precise manner (65, 66). Whether depending on the time course and the activating stimuli, platelets can enhance or inhibit tumor angiogenesis by selective release of pro- or anti-angiogenic factors is an intriguing question, which needs to be addressed in an *in vivo* context.

Tumor cells can secret VEGF and platelets isolated from cancer patients selectively uptake and store VEGF in α-granules. However, depending on the pathological conditions, cancer cells can also stimulate the release of platelet-resident VEGF, thereby regulating the local VEGF concentrations in the tumor microenvironment, which strongly influences tumor angiogenesis. Tumor-derived IL-6 increases VEGF expression in megakaryocytes, and consequent platelet VEGF levels also increased in platelet α-granules, suggesting a complex interplay between platelets and cancer cells in the regulation of tumor angiogenesis (67).

In tumor xenograft and ischemic hind limb hypoxia models, platelets promoted the homing of bone marrow-derived cells to the neovascularized hypoxic tissues. This process was dependent on platelet granule releseate, containing growth factors and cytokines (68). In the xenograft breast cancer model, platelets sequestered cytokines released by human luminal breast cancer cells and delivered them to indolent tumors, thereby ensuring tumor outgrowth during angiogenesis (69).

Tumor-derived VEGF mediates endothelial cell activation, vWF release, and platelet aggregation thereby provoking the coagulation cascade in patients with melanoma cancer (70). vWF release was accompanied by local inhibition of proteolytic activity and protein expression of vWF cleaving protease ADAMTS13, (a disintegrin-like and metalloproteinase with thrombospondin type I repeats 13). VEGF can also enhance TF expression in the endothelium and inflammatory cells, which further increases platelet adhesion and thrombin generation (71). Accumulation of activated platelets was also observed on the fibrinogen/fibrin-coated endothelial surface. In turn, this provisional fibrin matrix supports endothelial cell survival and migration (72). Interestingly, the platelet δ-granule-resident ADP, which was not critical for vessel formation, could facilitate endothelial cell migration (73).

Besides platelet-releseate, platelets possibly regulate angiogenesis independently of their granular content. Platelets can promote tube formation of HUVEC in a matrigel assay and this process was enhanced by direct contact between platelets and endothelial cells (74). Using a monoclonal antibody c7E3 Fab (abciximab, ReoPro), inhibiting integrin αIIbβ3 function on the platelet surface, the platelet-enhanced capillary formation was reduced during tumor growth, hypoxia-induced retinal angiogenesis, and also in HUVEC *in vitro* (75, 76). Recent studies demonstrated that platelet tetraspanin could promote endothelial colony-forming culture tube formation (77). Tetraspanin function is also linked to laminin-specific integrin α6β1, since blockade of this type of integrin in both platelets and endothelial cells could attenuate the ability of platelets to promote tube formation (77). In similar conditions, platelet CD154 and endothelium-resident CD40 receptors further enhance platelet aggregation and thrombus formation (71, 78).

Activated platelets shed PMPs which are vesicular fragments with a size of 0.05 to 1 μm. PMPs contain several receptors and proteins on the membrane surface, including P-selectin and integrins, and store many growth factors, cytokines and inflammatory molecules (79, 80). Elevated PMP levels were detected in the plasma of cancer patients (81). Interestingly, PMPs could induce angiogenic effects to a similar extent as whole platelets. Increasing levels of VEGF, IL-6 and Regulated on Activation Normal T Cell Expressed (RANTES) were detected in PMPs isolated from patients with gastric cancer suggesting that PMP-shedding contributes to tumor angiogenesis (82). Platelets and PMPs also contain different types of miRNAs. Transferring of PMP-specific miRNA let-7a or miR-27b into endothelial cells could inhibit the expression of TSP1, thereby enhancing platelet-dependent endothelial tube formation (83, 84).

### Lymphangiogenesis

Angiogenesis also occurs in lymphatic vessels, supporting tumor growth and dissemination. During embryonic development, platelets maintain the separated blood-lymphatic system by interacting with PDPN at the lympho-venous junction (85). In CLEC-2-deficient mice, lymphatic vessels were filled with blood, resulting in embryonic lethality (85). In the tumorigenesis model induced by intradermal injection of B16F10 melanoma cells, CLEC-2 deficiency was also associated with blood filling in lymphatic vessels (86). However, future studies are needed to evaluate the molecular mechanism, how platelet-resident CLEC-2 can regulate lymphatic vessel separation during tumorigenesis.

### Vasculogenic Mimicry

Vasculogenic mimicry occurs often in patients with aggressive cancer types, such as melanoma and cholangiosarcoma and possibly promotes tumor metastasis (87). Vascular mimicry reflects the ability of tumor or tumor stem cells to form vessel-like networks for the obtention of oxygen and essential nutrients independently of sprouting angiogenesis (54). Interestingly, in contrast to other types of angiogenesis, platelets inhibit vasculogenic mimicry, indicating that platelets tightly coordinate the vascularization process, and may still potentiate tumor malignancy (88).

### Tumor Vascular Integrity

Besides their pivotal role in angiogenesis, platelets regulate tumoral vascular integrity in primary tumors, thereby preventing tumor hemorrhages. Initial studies from Ho-Tin Noé et al. showed that the maintenance of tumor vascular integrity resulted in the secretion of platelet granular-resident serotonin and ANGPT, which stabilizes the structure of tumor blood vessels by counteracting with tumor-derived VEGF (89). By maintaining vascular integrity, platelets could reduce tissue damage, which was caused by tumor-infiltrating immune cells (90, 91). Later, it was proposed that tumor vessel destabilization might have beneficial effects, promoting effective delivery of chemotherapeutic agents into growing tumors (92). Recent studies showed that tumor vessel integrity depends on platelet-specific receptor GPVI and blocking this receptor in primary prostate and breast cancer tumors could increase the efficacy of chemotherapy (91). Although these studies highlighted the role of platelets and GPVI in the maintenance of tumor vessel integrity, the underlying molecular mechanisms remain elusive.

Depending on the disease context, platelets could regulate neovascularization, leading to the generation of permeable vessels. On the other hand, platelets could also induce vessel stabilization by maintaining vessel integrity, and in some cases, facilitated vessel maturation (93, 94). Compelling experimental and clinical pieces of evidence showed the heterogeneity and plasticity of the tumor and host cells. Growing tumors need a specific organization and structure of tumor vasculature. Platelets are the major cellular regulators of this process. Targeting tumor angiogenesis is an important concept in cancer research, which led to the development of anti-angiogenic therapeutic strategies. However, the beneficial effects of anti-angiogenic therapies are often limited, due to several factors expressed by the tumor, inducing diverse cell-type-specific resistance mechanisms (95). Hence, platelets regulate many functions of tumor vasculature, it is tempting to speculate that targeting platelet-mediated pathways in angiogenesis would be considered as an alternative anti-angiogenic strategy.

## Platelet Functions in Tumor Metastasis

**Figure 3:**
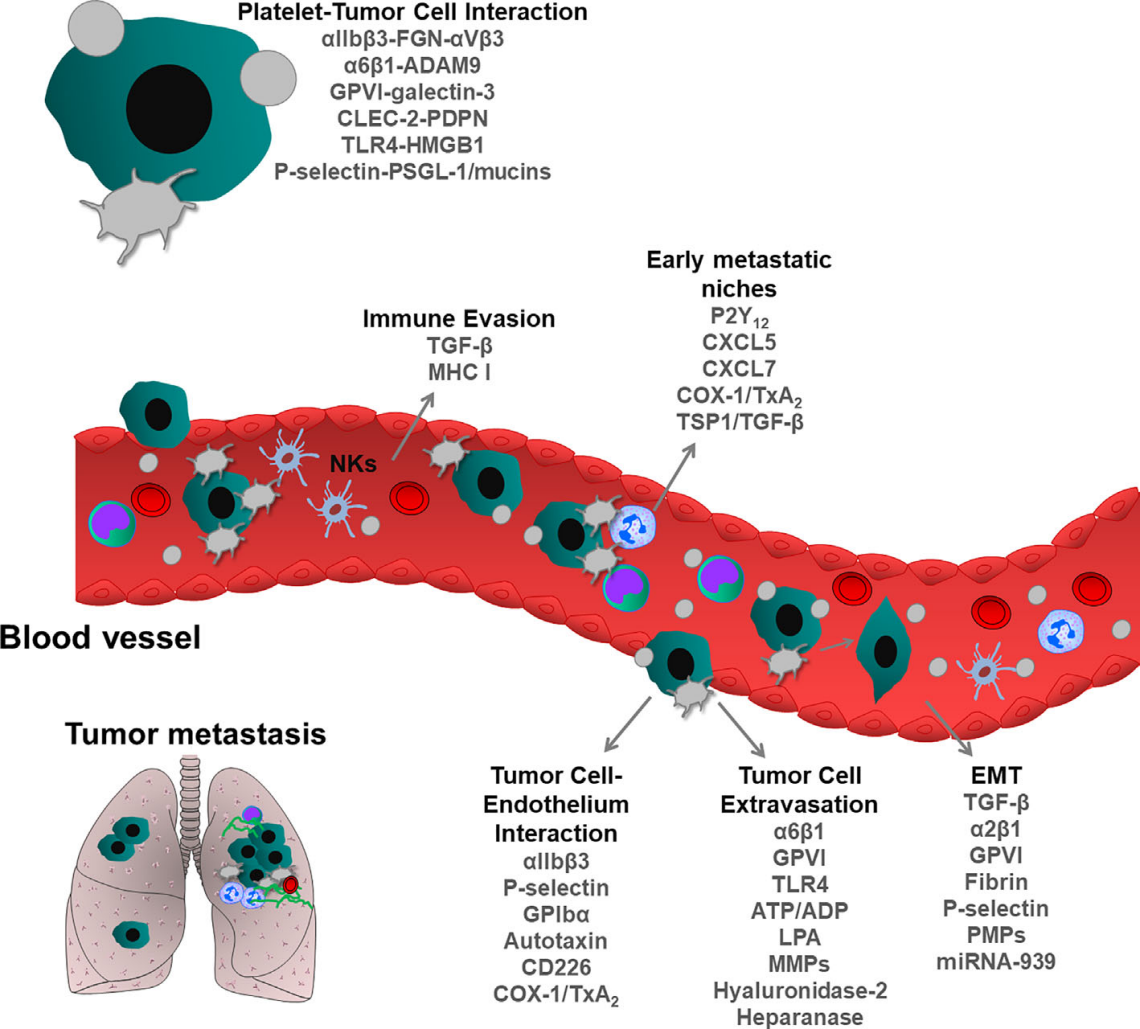
Role of blood platelets in tumor metastasis. During metastatic spread, tumor cells enter into the blood circulation where they encounter tumor cells. Physical and functional interactions between platelets and tumor cells support metastatic dissemination of tumor cells and survival at distant metastatic organs. Platelets protect tumor cells from immune system surveillance, thereby enhancing the survival of circulating tumor cells. Following interactions with platelets, epithelial cancer cells can acquire mesenchymal phenotype, rapidly invading distant organs. Platelets form an active complex with inflammatory and tumor cells within the vasculature, thereby supporting the formation of metastatic niches. Platelets also can directly bridge tumor cells to the endothelium and induce their extravasation, leading to the seeding and proliferation of metastatic cells. As shown, each step of metastatic cascade is regulated by platelet-resident receptors and or other platelet-derived bioactive molecules, such as growth factors, cytokines, chemokines and miRNA.

Invasive tumor cells that are detached from primary tumors can migrate and colonize and proliferate at distant sites, thereby forming secondary tumors, called metastases. Invasive cells entering into the vascular or lymphatic system undergo various shear and oxidative stress, also cytotoxic effects of immune cells, thereby reducing the number of cancer cells in the peripheral blood. However, a few numbers of tumor cells can escape from these processes, extravasate from vessels and colonize distant organs (96, 97). During this transit in the circulation, platelets are the first cells that encounter tumor cells, thereby supporting their metastatic potential. Several molecular mechanisms have been described in platelet-mediated steps of tumor metastasis (**Figure 3**). First experimental evidence was described by Gasic et al., who showed that thrombocytopenia was correlated with reduced metastasis and transfusion of platelets into the thrombocytopenic mice restored the capacity to form metastases (98). In line with this, later studies described that impaired megakaryopoiesis and platelet production also reduce metastatic events in different mouse models (99).

### Platelets and Immune Evasion of Cancer Cells

From all the cancer cells that enter into the circulation, only a small number forms metastatic foci (96, 100). Besides shear stress, cytotoxic natural killer (NK) cells eliminate most cancer cells from circulation. Platelets are the first blood cells to interact with cancer cells and serve as a physical ring to protect cancer cells from immune system surveillance (96). Studies inducing thrombocytopenia in mice suggest that platelets impact NK cell-mediated lysis of tumor cells (101) and this hypothesis was followed by Palumbo et al., using fibrinogen or Gαq protein-deficient mice and their mutant platelets (102). In both cases, cancer cell survival was strongly reduced. These studies proposed that activated platelets together with fibrinogen (or fibrin) could cover cancer cells and protected them from NK cell-induced killing mechanisms (102). However, others showed that platelets exert their pro-metastatic effects within the first hour following the entry of tumor cells into the circulation, whereas anti-metastatic effects of NK occur between 1 and 6 hours after tumor cell intravasation (103). During interactions with tumor cells, platelets transfer major histocompatibility complex (MHC) class I molecules on tumor cells, thereby providing a self-signal to NK cells to inhibit their killing activity *in vitro* (104). In addition, platelet-mediated shedding of Natural Killer Group 2D (NKG2D) ligands could also inhibit this cytotoxic effect of NK cells (105). Platelets can store a significant amount of TGF-β in α-granules (50-100 times more than other blood cells) (106), and release it into the circulatory system (107) and tumor microenvironment (108) during cancer progression and metastasis. It has been shown that platelet-derived TGF-β causes the downregulation of NKG2D on NKs upon interaction with cancer cells, inhibiting anti-tumor immunity (109). In line with this, the downregulation of NKG2D has been associated with elevated levels of TGF-β in patients with colorectal and lung cancer (110). TGF-β-docking receptor glycoprotein A repetitions predominant (GARP) protein activates the latent form of platelet TGF-β and this protein complex together with platelet-secreted lactate inhibited immune response against both melanoma and colon cancer (108). Using the TGF-β-GARP complex, platelets could directly inhibit T cell function *in vitro* and *in vivo* experimental conditions, and TGF-β rich platelet releasate and lactate can suppress both CD4+ and CD8+ T cell activity (108). Interestingly, thrombin can contribute to immune evasion by cleaving platelet-bound GARP thereby activating latent TGF-β (111). TGF-β inhibitors, blocking peptides and aptamers are currently used in clinical trials of patients with solid cancers (112). Using adoptive T cell transfer, as a therapeutic approach to stimulate the immune system, has been proposed as a promising anti-cancer strategy (113). It worth postulating that inhibition of TGF-β uptake by platelets could be a potential anti-cancer strategy enhancing protective immune pathways.

### Platelets and Cancer Cell Reprogramming

Epithelial-mesenchymal transition (EMT) is an important developmental program, which can also occur during cancer progression. Epithelial cancer cells change their morphology as they lose contact with the basement membrane and form the primary mesenchymal cell layer through EMT (114). Interestingly, the process of EMT can be reversible and primary mesenchymal cells can also be transformed into epithelial cells, and vice versa. EMT is supported by immune and stromal cells, also cells derived from the tumor microenvironment as well as the component of the ECM (114). Numerous factors are involved in the regulation of EMT, such as TGF-β, Hepatocyte Growth Factor (HGF) and EGF receptor, and transcription factors (ZEB1/2, Snail, Twist and Tiam1). Interestingly, EMT-like events occur during the intravascular transit of cancer cells when platelets interact with them, and at this moment, platelets release EMT inducers. In platelet-treated cancer cells, mesenchymal markers Snail family transcriptional repressor-1, vimentin, N-cadherin, fibronectin and matrix metalloproteinase 2 (MMP-2) are frequently upregulated, while the epithelial markers (E-cadherin, claudin-1) are downregulated (107). Activated platelets can release TGF-β from α-granules, switching cancer cells to a pro-metastatic EMT phenotype. Platelet-derived TGF-β and platelet-tumor cell interaction activate the TGF-β and nuclear factor kappa light chain enhancer of activated B cells (NF-κB) pathways in cancer cells, resulting in EMT phenotype and enhanced metastasis *in vivo*. In line with this, inhibition of NF-κB signaling in cancer cells or ablation of TGF-β expression only in platelets reduces metastasis in the lung (107). Altogether, these results suggest a direct link between platelet-resident TGF-β and EMT formation (107). Recently, tumor necrosis factor receptor-associated factor (TRAF) family member-associated NF-κB activator (TANK)-binding kinase 1 (TBK1) was identified as a mediator of platelet-induced EMT. Downregulation of TBK1 expression in cancer cells impaired platelet-induced EMT, which is due to the suppression of NF-κB signaling, suggesting that platelet-derived factors induce EMT in synergy with NF-κB pathways (115). This regulation is quite complex since platelets can induce EMT independently of NF-κB pathways. In ovarian cancer cells, platelet-derived TGF-β increased the invasive potential of tumor cells and induced EMT by increasing the phosphorylation of SMAD Family Member 2 (Smad2) (116).

ECM components, which are secreted by either tumor or tumor surrounding microenvironment, were proposed to be involved in EMT. Collagen and heat shock protein 47 (Hsp47), a chaperone that facilitates collagen secretion and deposition, was found to be highly expressed during EMT (117). Hsp47 expression induced mesenchymal phenotypes and enhanced platelet accumulation, leading to lung retention and colonization of cancer cells. Platelet depletion could abolish Hsp47-induced cancer cell retention in the lung, suggesting that Hsp47 promotes cancer cell colonization by enhancing cancer cell-platelet interaction (117). Accordingly, blockade of collagen receptor GPVI and integrin α2β1 on the platelet surface could abolish Hsp47-induced platelet interaction with 4T1, MDA-MB-231 and MCF10A breast cancer cells, indicating an important role of these platelet receptors in EMT (117). When MCF7 breast cancer cells were cocultured with platelets, EMT formation was also observed, and this process was mediated through direct contact with cancer-resident α2β1 integrin, leading to the activation of the Wnt-β-catenin signaling pathway (118). During systemic inflammation, platelet-fibrin-rich extravascular environment strongly activates intestinal inflammatory cells through αMβ2 integrin and this interaction can support the release of different cytokines and growth factors thereby further supporting EMT (119).

Cathepsin family members are proteases that are highly upregulated and secreted by different cancer cells. Cathepsins are mainly localized in endosomal or lysosomal vesicles and are also secreted, as soluble exo-enzymes, cleaving ECM components around the cancer cells, and activate or inactivate surface receptors by proteolytic cleavage (120). Interestingly, cathepsin K isoform could induce platelet aggregation, and supporting interaction with EMT-like cancer cells. This action is triggered by cathepsin-mediated PAR-receptor cleavage on the platelet surface (121). Hetero-aggregation of platelets with cancer cells induces P-selectin exposure and CD44 activation, which further enhances Hedgehog (Hdg)-signaling and also activates growth factors such as sonic hedgehog (SHH), osteopontin (OPN), parathyroid hormone-related protein (PTHrP), and TGF-β (121). PMPs also modulate EMT. Coculture of ovarian cancer cells with PMPs increases EMT (122). Overexpression or knockdown of PMP-specific miRNA-939 strongly enhances or inhibits EMT, respectively (122). PMP/miRNA-939 uptake by cancer cells was regulated by secretory phospholipase A2 type IIA (sPLA2-IIA), suggesting an important role for PMPs in the crosstalk between platelets and cancer cells during EMT (122).

### Platelet-Mediated Interactions of Circulating Tumor Cells With Vessels

Platelets play an important role in cancer cell trapping to vascular endothelium, thereby enhancing extravasation and dissemination to distant organs. Cancer cells are rolling along the endothelium, and this movement is maintained by platelet integrin αIIbβ3 and P-selectin to form stationary adhesion (123). Consequently, genetic deficiency or blockade of β3 integrin and P-selectin decreases cancer cell colonization in the lung (124, 125). P-selectin also binds mucins and P-selectin glycoprotein ligand-1 (PSGL-1) on the surface of tumor cells, mediating the interactions among platelets, leukocytes and endothelium (126, 127).

It has been hypothesized that exposure of vWF on the activated endothelial cell surface could also support the recruitment of platelet-cancer cell aggregates since genetic deficiency or antibody-mediated blockade of GPIbα, a vWF binding receptor on the platelet surface could inhibit TCIPA, also platelet-tumor cell interactions with endothelial cells, and consequent lung metastasis (128, 129).

Tumor cell-resident integrin αVβ3 also supports tumor cell interactions with platelets, allowing the interaction with vasculature and inducing tumor metastasis (130–132). At the invasive edge of tumor cells, αVβ3 integrins colocalize with nectin-like molecule 5 (NECL5). NECL5 interacts with CD226 on the platelet surface, enabling tumor cell adhesion to the endothelial vasculature, thereby leading to tumor metastasis (132). αVβ3 integrin on breast cancer cells can bind to platelet-derived autotaxin which is stored in α-granules and secreted into the vasculature upon platelet activation (133, 134). This process could induce early bone colonization and the progression of skeletal metastases in mice.

Although several tumor cell types adhere to the endothelium together with platelet aggregates and rapidly form thrombi, other cancer cells do not need platelets for increasing adhesion properties to the endothelium. Different models exist to group experimental pieces of evidence and explain platelet-dependent or -independent cancer cell adhesion. Cancer cells are rarely associated with platelets in the liver, rather directly bind the ECM, exposed by the discontinuous endothelium of the sinusoids (135, 136). Cell adhesion of leukemia cells was also independent of platelets and in the arterioles of pulmonary endothelium, thrombus formation was only observed at the later stage of metastasis (137). In other cases, treatment of mice with the thrombin inhibitor hirudin disrupted the interaction between cancer cells and platelets in the circulation, but this treatment did not affect cancer cell adhesion on the endothelium (138). Altogether, platelet-cancer cell interaction may support the intravascular arrest of cancer cells, thereby promoting rolling and static adhesion on the endothelium. However, the importance of platelet-cancer cell interactions with the vasculature strongly depends on the type of cancer cells and metastatic organs.

### Platelets in Dynamics of Tumor Cell Extravasation

After cancer cell arrest in the microvasculature, tumor cells transmigrate through the endothelium to form metastases. Most of the cancer cells attack capillaries and arterioles during extravasation. The structural differences of these vessels determine cancer cell behavior to rupture vessel barriers and successfully extravasate the targeted organs (139). Therefore, it is important to understand the distinct molecular mechanisms of extravasation related to vessel types, thereby designing selective therapeutic approaches to prevent metastasis. Several models exist to explain this complex pathological process, (i) how cancer cells and platelets modify vascular permeability, and (ii) how the endothelial cell layer and the basal membrane are ruptured, thereby promoting cancer cell extravasation. Platelets may promote endothelial cell retraction and paracellular migration of tumor cells, although in many cases, this process can be regulated independently of platelets (6). Platelets can damage the endothelium layer, releasing inducers of necroptosis (140), also damaging ECM network, thereby enhancing the extravasation of the cancer cells. Here, we highlight some platelet-derived factors that contribute to different stages of cancer cell extravasation.

Molecules stored in α- and δ-granules can regulate vascular permeability. Upon activation, degranulated platelets release serotonin, VEGF, platelet-activating factor (PAF), thrombin, ATP/ADP, HGF, fibrinogen, which can potentially induce vascular permeability, and consequently promote cancer cell transmigration (71, 97). Studies showed that the extravasation of cancer cells into the lung parenchyma was reduced in Unc13d (Munc-13-4) knockout mice, in which platelet δ-granule secretion was severely, and α-granule secretion was moderately inhibited (141). Platelet-derived ATP induces the relaxation of endothelial junctions and vascular permeability by activating P2Y_2_ purinergic receptors on the surface of endothelial cells, and this process consequently was inhibited in P2Y_2_ knockout mice (141). Recently, we identified a novel molecular mechanism that further supports ATP-mediated effects in cancer cell metastasis. We showed that galectin-3 expressed on the surface of colon and breast cancer cells interacted with platelet-specific receptor GPVI and this receptor-ligand mediated cellular crosstalk could enhance platelet activation and degranulation (ATP secretion) which further support tumor cell extravasation (142). In line with previous studies, impaired δ-granule release and apyrase-mediated blockade of ATP function in platelets, strongly inhibited endothelial transmigration of tumor cells, indicating an important role of platelet-derived nucleotides in this process (141, 142).

Serotonin is a biogenic monoamine produced from tryptophan. Serotonin synthesis takes place in the enterochromaffin cells, located in the gastrointestinal tract, that release serotonin into the blood, where platelets rapidly take it up into δ-granules (143). Local accumulation of serotonin modulates the vascular tone thereby influencing shear-dependent processes. Circulating tumor cells increase the plasma levels of serotonin and blockade of serotonin receptors or calcium channels could effectively inhibit experimental liver metastasis, indicating multiple roles of serotonin in different steps of cancer progression (144). However, the role of platelet-derived serotonin in tumor progression, including the extravasation stage, has not been investigated.

Activated platelets release lysophosphatidic acid (LPA) from their α-granules, which can also induce tumor cell invasion and influence the permeability of the endothelium, thereby promoting transendothelial cell migration (145, 146). In line with this, lung metastasis was impaired in Neurobeachin-like 2 (Nbeal2) knock-out mice lacking α-granules (147). Blocking platelet activation leads to a decrease in LPA concentration in the blood. Autotaxin is a secreted glycosylated enzyme with lysophospholipase D (LPD) activity, which is responsible for the regulation of basal LPA levels in the blood (148). Interestingly, cancer cell-resident CD97/G-protein-coupled receptors (GPCR) can induce platelet activation thereby enhancing both LPA release from α-granules and ATP secretion from δ-granules (145). Consequently, this platelet releseate could increase tumor cell-induced vascular permeability and lung metastasis (145).

ADAM9 is a member of the disintegrin and metalloproteinase (ADAM) family, which induce the shedding of receptors (149). Interestingly, this proteinase can promote tumor cell migration and metastasis in both MMP-dependent and -independent manner (149–151). Interaction of platelet-resident α6β1 with the disintegrin-cysteine-rich domain of tumor cell-derived ADAM9 also triggers platelet activation, α-granule release and P-selectin exposure, increasing tumor cell extravasation (150). Platelet-tumor cell interactions mediated by platelet toll-like receptor 4 (TLR4) and tumor cell-released high-mobility group box 1 protein (HMGB1) lead also to the α-granule release and P-selectin exposure, subsequently increasing the number of extravasating tumor cells in lung vessels (152).

To effectively cross the subendothelial layer, cancer cells should damage and move through the basement membrane. Platelets can store and release several exo-enzymes, such as MMPs, platelet hyaluronidase-2, and heparanase, that can degrade collagen-rich ECM components (153, 154). After platelet depletion, the reduced extracellular activity of MMPs was observed and consequently, the number of lung metastases was also reduced in mice, highlighting the contribution of platelet-derived MMPs in the degradation of the basement membrane (155).

Altogether, these studies suggest that platelets are involved in tumor cell extravasation, although only limited *in vivo* experiments showed the importance of platelet receptors and enzymes in the process of basement membrane degradation.

### Platelets and Metastatic Seeding

During systemic inflammation, the release of ECM stimulates the environment of distant organs to accept tumor cell seeding, thereby actively enhancing tumor metastasis (156). Once tumor cells metastasized and formed certain tumor mass in host organs, evolved metastatic niches can again contribute to the recruitment of circulating blood and inflammatory immune cells (156, 157). In different steps of metastatic niche formation platelets are involved: (i) platelets can support cancer cell adhesion and granulocyte recruitment in the early metastatic niche, (ii) platelets can release different chemokines which stimulate the recruitment of host cells to build the tumor microenvironment, (iii) platelets also release pro-angiogenic factors at a later stage to induce local tumor vessel formation within the host microenvironment, (iv) platelets create an immune cell-rich environment around the developing metastases thereby supporting tumor cell proliferation and survival.

Growing tumors release angiogenic growth factors, allowing cancer cells to develop a niche before they metastasize. Tumor cell-derived VEGF alters the microenvironment of distant organs. VEGF triggers inflammation, also increases cyclooxygenase (COX) products and PGE2, leading to the homing of cancer cells to the lungs (158). Several ECM components, integrins and VEGF receptors have been identified as main regulators of organ-specific cancer cell tropism and niche formation (159). In line with this, platelet ADP receptor P2Y_12_ induces the recruitment of VEGFR1+ bone marrow-derived cell clusters and fibronectin deposition, which creates a premetastatic niche, selectively promoting lung metastasis (160).

During tumor metastasis, monocytes and macrophages are recruited to the metastatic niches and support cancer cell seeding. Platelet-resident chemokines (CXC motif ligand (CXCL) 5 and CXCL7) have been shown to promote the early stage of the metastatic niche through activation of granulocyte-derived C-X-C chemokine receptor 2 (CXCR2) (161). When cancer cells interact with platelets, chemokines from platelet α-granules are released which recruit granulocytes to the cancer-platelet aggregates thereby supporting the seeding of metastatic cells (161).

Fibrin-rich platelet aggregates also provide a provisory matrix that further supports metastatic seeding. Indeed, TF expressed in brain cancer cells induces the coagulation cascade that results in thrombin formation, subsequent platelet activation and fibrin formation (162). Impairment of macrophage function in Mac- or CD11-deficient mouse models inhibits tumor cell survival, suggesting that the recruitment of functional macrophages to the platelet-rich clots is essential for this process (162). TxA_2_ stimulates macrophage infiltration and cytokine release (163). Using an experimental metastasis model with B16F10 melanoma cells, Lucotti et al. showed that the platelet-specific COX-1/TxA_2_ pathway induces platelet-tumor cell aggregation, endothelial activation, tumor cell adhesion to the endothelium, and also recruitment of monocytes/macrophages, thereby promoting premetastatic niche formation in the lung (164).

On the other hand, depending on the developmental phase of cancer and environmental stimuli, immune cells and granulocytes could also induce cell death in metastatic cancer cells. Derivates of PC3 prostate and MDA-MB-231 breast cancer cells with poor metastatic capacity can recruit pro-metastatic Gr+ myeloid cells and generate a metastasis-refractory microenvironment by inducing the secretion of TSP1, which seems to inhibit lung metastasis (165). In contrast, platelet-resident TSP1 had the opposite effect on bone metastasis. Within the bone microenvironment, TSP1/TGF-β axis is involved in the regulation of premetastatic niche formation and bone metastasis (166).

Therapeutic targeting of early stages of cancer, evolving metastatic soil, is important in cancer patients. It could be interesting to evaluate the platelet-derived protein signature and the presence of platelet-tumor-immune cell conjugates in cancer patient tissues can represent prognostic and diagnostic tools, thereby helping an earlier intervention.

## Therapeutic Targets

Platelet adhesion, activation, and aggregation are tightly regulated at different steps of tumor progression, thereby influencing the coagulation cascade and thrombus formation in cancer patients. Integrins, glycoproteins, and many other signaling receptors on the platelet surface are involved in these processes. Below, we discuss the therapeutic effects on the function of the main platelet receptors and regulatory mechanisms controlling tumor progression and metastasis.

### Integrins

Platelets express different integrins, including α2β1, α5β1 and α6β1, to facilitate the binding to collagen, fibronectin and laminin, respectively. Integrin α2β1 together with GPVI mediates direct interactions of platelets with collagen, exposed by the subendothelial matrix (167). Although it has been shown that blockade of α6β1 integrin on platelets could abolish platelet-tumor cell interaction and tumor metastasis (150), *in vivo* functions of other types of β1 integrins have not been established.

In tumor metastasis models injecting B16F10 melanoma, AT-3 breast or MC38 colon cancer cells, we showed that platelet integrin α6β1 promotes metastasis through the binding to the tumor cell-derived ADAM9 (150). Blockade of integrin α6 functions with GoH3 antibody could inhibit platelet-tumor cell interaction *in vitro* and tumor metastasis *in vivo* (150). Genetic or antibody-mediated blockade of α6 functions in mice did not alter hemostasis or platelet numbers (150). Remarkably, this antibody had no additive effects on tumor metastasis when administered intravenously to platelet α6β1-deficient mice, at the same time as tumor cells, indicating a predominant role of this platelet integrin during the early steps of tumor metastasis (150). Of note, α6β1 is also detected in other cells, such as cancer and endothelial cells, and pericytes, transferring pro-tumorigenic effects into the tumor environment (168). Targeting α6β1 integrin inhibits several routes of integrin-mediated tumor malignancy, therefore blockade of their functions may be a safe anti-cancer strategy. Recent studies by De Archangelis et al. showed that α6 integrin deficiency in mouse intestinal epithelial cells resulted in disruption of hemidesmosome integrity, and mice developed colitis and colorectal carcinoma (169). Integrin α6 deficiency in mice and humans also caused skin and mucous disorders, such as pyloric atresia and epidermolysis bullosa (170). Therefore, determining the *in vivo* side-effects of α6β1 blockade is necessary before the therapeutic implication.

Integrin αIIbβ3 is the major platelet integrin with a high copy number on the surface, switching from an inactive to an active ligand-binding conformation after agonist stimulation thereby regulating platelet aggregation, thrombosis and hemostasis (171). This transformation allows αIIbβ3 integrin to bind fibrinogen and vWF and those bridge platelets together (8, 171). Antagonists of αIIbβ3 integrin already used in the treatment of patients with acute coronary diseases (172). Integrillin, a potent αIIbβ3 integrin blocker, could effectively inhibit TCIPA and breast cancer-associated bone metastasis (173). Consistently, the genetic ablation of β3 integrins in mice inhibited bone metastasis (124). Of note, deficiency of αIIb subunit in mice also decreased early steps of lung metastasis, but surprisingly opposite effects were observed at later stages of metastasis (174). The expression of αIIbβ3 integrin is not exclusive to platelets, it is also detected on the surface of breast cancer cells (175). In addition, integrillin inhibits αvβ3 integrin function (176), and this integrin was also detected in cancer and endothelial cells, macrophages, and at low levels on the platelet surface (168), Therefore, blockade of β3 integrin function has been suggested as a beneficial therapeutic approach against tumor cells within their environment. However, blockade of αIIbβ3 integrin functions in cancer therapy can result in serious side-effects on hemostasis, causing bleeding complications with life-threatening consequences. A possible compromise would be to develop more specific inhibitors that only target the active form of αIIbβ3 integrins, reducing the risk of bleeding complications. Recently, several groups have been proposed an alternative strategy to prove this concept, and also partially delete αIIbβ3 integrin functions. An antibody-based strategy blocking the thiol-isomerase function of αIIbβ3 integrins could inhibit thrombosis without inducing bleeding (177, 178). Alternatively, using a single-chain antibody (ScFv) directed against the active form of αIIbβ3 integrin, similar anti-thrombotic effects were observed without any hemostatic complication (179). In line with this, in human cancer xenograft models (breast: MDA-MB-231, SKBr3; fibrosarcoma: HT-1080; Burkitt’s lymphoma: Ramos) ScFv antibody effectively targeted the active form of platelet αIIbβ3 integrins (180). Recently, an antibody-drug conjugate (ADC) was developed, linking ScFv to a potent chemotherapeutic microtubule inhibitor monomethyl auristatin E (MMAE), against the active form of platelet αIIbβ3 integrins and simultaneusly blocking tumor growth and metastasis by MMAE (181). In the tumor microenvironment, MMAE is cleaved from the ADC by cathepsin B, thereby locally releasing the bioactive form of MMAE, killing cancer cells without bleeding complications (181). This promising therapeutic tool would be important to follow in the future using immunocompetent mouse models of cancer.

### GPIb-V-IX Complex

Glycoprotein (GP)Ib-V-IX complex regulates platelet adhesion to the injured sites of a vessel, and platelet aggregation, particularly under the condition of high shear stress (8). This receptor complex is composed of four membrane glycoproteins: GPIbα, GPIbβ, GPIX and GPV (8). Using GPIbα/IL4R transgenic mice, replacing the extracellular domains of GPIbα to IL4R, Jain et al. showed that deletion of vWF binding and other binding sites located in these domains of GPIbα could inhibit experimental lung metastasis which was induced by B16F10-melanoma cells (128). Later studies by Erpenbeck et al., showed that treatment with a mixture of GPIbα antibodies (p0p3 and p0p4) directed against vWF binding site on GPIbα could also inhibit tumor metastasis using a similar mouse model of lung metastasis (182). However, p0p3/p0p4 antibodies against GPIbα induced severe thrombocytopenia (182), which probably accounts for this inhibitory effect. Surprisingly, another GPIbα antibody (p0p/B Fab fragment) enhanced tumor metastasis in this study (182). Although p0p/B Fab was described to block vWF and thrombin binding to mouse GPIbα and the functional blockade of GPIbα with p0p/B Fab was proved using different platelet functional tests (183, 184), the binding epitope on GPIbα has not been mapped at a biochemical level. Taken these results together, it is difficult to conclude at the moment which step of the metastatic cascade is regulated by GPIbα. Nevertheless, mice treated with p0p/B Fab displayed prolonged tail-bleeding times (185), indicating a major drawback of such antibody treatment in experimental cancer models.

Recently, the role of GPIbα was further analyzed in experimental (Lewis lung carcinoma) and spontaneous (4T1 breast cancer) lung metastasis models using a YQ3 antibody which specifically inhibits GPIbα-vWF interaction (129). The authors showed that platelet-tumor cell and platelet-endothelial cell interactions, and TCIPA were inhibited *in vitro* and also lung metastasis *in vivo* (129). Control experiments showed that the Fab fragment of YQ3 antibody did not induce platelet activation or clearance, or any off-target effects when it was injected into the GPIbα/IL4R transgenic mice, indicating that this blocking strategy can avoid thrombocytopenia or bleeding complications in cancer mouse models (129).

GPIbα function was also studied in the disease context of inflammation-related carcinogenesis. Platelet-Kupffer cell interaction involves hyaluronan-CD44 binding and early platelet activation in the liver, which contributes to non-alcoholic steatohepatitis (NASH)-associated liver carcinogenesis (186). Genetic deficiency or blockade of GPIbα function using p0p6 antibody, which was earlier described as GPIbα and GPIX inhibitor (186, 187), suppressed NASH-inducing pathological effects, and this process was independent of the interactions of GPIbα with vWF, P-selectin, or αMβ2 integrin (186). GPIbα blockade is known to inhibit TPO production in the liver. Theoretically, this may influence consequent platelet production and response to TCIPA in this cancer model (188). Future studies are required to test this hypothesis.

A snake venom-derived antagonist anfibatide and humanized anti-GPIbα monoclonal antibody h6B4-Fab have been also proposed as safe antithrombotic agents in different preclinical and clinical studies (189–191). In the future, it would be important to study their functions also in similar mouse models of cancer as mentioned above.

Besides GPIbα specific antibodies, GPIbβ blocking antibody RAM.1 has been tested *in vivo* thrombosis models (192). Although RAM.1 treatment had strong anti-thrombotic effects, bleeding times were not affected in mice, excluding major impact on hemostasis (192). However, the physiological role of GPIbβ and other subunits of the receptor complex (GPV and GPIX) have not been further studied in mouse models of cancer.

### Glycoprotein VI

Glycoprotein VI (GPVI) is a receptor of ITAM-signaling, which is activated with collagen, laminin and fibrin (193). Activation of this receptor regulates diverse physiological processes in platelets, including adhesion, activation, aggregation and pro-coagulant activity. Although the role of GPVI function in cancer metastasis has been investigated by injecting Lewis lung carcinoma or B16F10 melanoma cells into mice (194), only limited studies explained the exact molecular mechanisms of how platelet GPVI contributes to this process. A soluble form of GPVI, Revacept has been developed and evaluated in clinical trials of patients with thrombotic diseases to disrupt the interaction of platelet-resident GPVI from collagen thereby inhibiting thrombus formation (195, 196). Using galectin-3-expressing HT29 cells, Dovizio et al. showed that Revacept had an inhibitory effect on COX-2 and platelet-mediated EMT *in vitro* (197). Recently, we showed that GPVI supports platelet adhesion on colon and breast cancer cells. We identified galectin-3 on the surface of these cancer cells and proposed a model that the collagen-like domain of galectin-3 interacts with GPVI (142). This interaction triggered platelet activation and subsequent extravasation of tumor cells, leading to tumor metastasis (142). Using *in vitro* and *in vivo* models, we showed that the blockade of this interaction could prevent platelet-tumor cell interactions and inhibited colon and breast cancer cell-associated lung metastasis in mice (142). The effect of galectin-3 can be shared by other members of the galectin family, such as galectin-1 and galectin-8. Both of these molecules can induce platelet activation (198) and the release of pro-angiogenic molecules that enhance HUVEC angiogenic responses including proliferation and *in vitro* tubule-like formation (199).

Besides collagen, GPVI can also bind other ECM components, such as fibrin, linking its function to the coagulation cascade and fibrin-dependent pathomechanisms (200–202). Fibrin-GPVI interactions contribute to thrombus growth (200, 201), which can also occur in patients with cancer, leading to thromboembolic events. Therefore, it would be necessary to further investigate the role of GPVI in the context of fibrin-dependent cancer progression and metastasis. Only abrogating fibrin binding to GPVI would be important to evaluate in the future and find the distinct role of fibrin-GPVI interaction in tumor malignancy and cancer-induced coagulopathy. Of note, GPVI also interacts with many ECM components and adhesion molecules, such as fibronectin, vitronectin, adiponectin, MMP13, EMMPRIN and histones (193, 203). Therefore, we proposed that GPVI-mediated platelet-tumor cell interactions would be cell type-specific, and it may also depend on the repertoire of various ligands, which are overexpressed in different cancer cells. Furthermore, we also showed that genetic deficiency or antibody-mediated inhibition of GPVI can induce intratumoral hemorrhage, thereby increasing the efficacy of chemotherapeutic drugs within the prostate and mammary tumors (91). Although this has been observed in two distinct cancer models, the treatment of humanized GPVI mouse model with B16F10 skin tumor and blocking human GPVI function with Glenzocimab (ACT017) did not cause tumor bleeding (204). Depending on the cancer types, tumor progression and vascularization seem to be regulated by different oncogenic signaling pathways that modify the hemostatic effects of GPVI.

GPVI has been considered as a potentially safe anti-thrombotic target based on the observations that its signaling blockade reduces experimental thrombosis without impairing hemostasis (193). GPVI is exclusively expressed in platelet/megakaryocyte lineage. Antibody blockade or genetic deficiency of GPVI does not influence platelet production or hemostasis, neither in mice nor in humans (193, 205). Altogether, these results strongly indicate that therapeutic strategies based on selective blockade of GPVI function or its interactions with the indicated ligands in some cancer types may blaze the trail for new anti-cancer therapies, preserving normal hemostasis.

### C-Type Lectin-Like Receptor-2

C-type lectin-like receptor-2 (CLEC-2) has a restricted expression pattern in humans, mainly detected in megakaryocytes, platelets, dendritic cells, and Kupffer cells. Genetic or pharmacological blockade of CLEC-2 in mice does not influence platelet production or hemostasis. Therefore, CLEC-2 would be an attractive target for cancer therapy (33). Recently, targeting CLEC-2 function in cancer was proposed to be effective in decreasing hematogenous cancer metastasis, cancer-associated thrombus formation and thrombo-inflammation (33). Injection of B16F10 melanoma cancer cells into the back skin of CLEC-2-depleted mice followed by injection of the 2A2B10 monoclonal antibody inhibited thrombus formation in the tumor vessels without developing intratumoral hemorrhages (206). However, the functional vessel density was significantly increased in CLEC‐2-depleted mice, improving oxygen and nutrient supply in the tumor environment, indirectly promoting tumor proliferation (206). These interesting pathological aspects of CLEC-2 deficiency opens the question of whether pharmacological blockade of CLEC-2 function could be beneficial in cancer patients.

Interestingly, aberrant O-glycosylation was detected on cancer-origin PDPN. The LpMab-2 antibody recognized this specific site and effectively inhibits PDPN-CLEC-2 interaction only in the cancer microenvironment (207). Therefore, it was proposed that an LpMab-2 antibody would be an excellent tool for selectively targeting PDPN-positive cancer cells, inhibiting cancer-related thrombosis within tumor vessels, without interfering with normal cells that are located in lymphatic vessels (207). Other functional blocking monoclonal antibody (mAb, SZ168) against the extracellular domain of human PDPN also significantly inhibited the growth and pulmonary metastasis of human malignant melanoma (208). Of note, simultaneously blocking both CLEC-2 and GPVI receptor functions may cause severe bleeding complications, as it was demonstrated in CLEC-2/GPVI-depleted mice (209).

Platelet aggregation-inducing domains (PLAGs) of PDPN were recently identified, linking its function to CLEC-2. Using anti-PLAG-neutralizing antibodies, the function of PLAGs was confirmed in CLEC-2 binding, platelet aggregation, and tumor emboli formation indicating that simultaneous inhibition of PLAGs is efficient to block PDPN-mediated tumor growth and metastasis (210). Besides blocking antibodies, several CLEC-2-binding small molecules can interrupt CLEC-2-PDPN interaction. The water extract of Chinese medicine from leaves of Artemisia argyi could irreversibly block CLEC-2-PDPN interaction in a dose-dependent manner (211). This action may result in the prevention of tumor metastases. Another drug, 5-nitrobenzoate-2CP also inhibits PDPN-CLEC2 interaction and consequent TCIPA formation without a risk of bleeding (211). Therefore, it was proposed that inhibition of PDPN-CLEC-2-mediated TCIPA may provide effective therapy against metastasis and thromboembolic complications. The modified form of snake venom toxin rhodocytin was also proposed as a potential tool to target CLEC-2 for both anti-platelet and anti-metastatic therapy (212, 213).

Altogether, these results suggest that selective blockade of CLEC-2 function on the platelet surface or disruption of PDPN-CLEC-2 interaction would be effective for anti-cancer therapy. Further studies are required using different experimental cancer models and humanized CLEC-2 mice before clinical application.

## Cyclooxygenases

Upon platelet activation, thromboxane A_2_ (TxA_2_) secretion enhances platelet aggregation and thrombus formation through its thromboxane-receptor (TP) and also induces diverse paracrine effects on neighboring cells in thrombotic and tumorigenic conditions (214). Aspirin (acetylsalicylic acid) irreversibly inhibits the enzymatic activity of cyclooxygenases (COXs) which are involved in arachidonic acid metabolism, producing TxA_2_ (172). Aspirin binding covalently modifies COX-1 and COX-2 isoforms through acetylation of serine residues 529 and 516, respectively (215, 216). Although platelets constitutively express COX-1, COX-2 expression is dramatically upregulated during inflammatory or tumorigenic conditions. Increased COX-2 expression was detected in many cancer types, such as breast, bladder lung, pancreatic, gastric and lung (217, 218).

An inhibitory role of aspirin in cancer development was first reported by Gasic et al. in 1973, who observed metastatic inhibition of MCA6 ascites sarcoma cells in aspirin-treated mice (219). Later, many *in vitro* and preclinical studies analyzed platelet-dependent effects of aspirin on cancer. Pretreated platelets with aspirin could effectively inhibit cancer cell-induced platelet aggregation (220), but in another study this treatment did not inhibit platelet degranulation, adhesion or micro-aggregate formation (221). These processes are possibly triggered by purinergic signalings which are not inhibited by aspirin in platelets (222).

The effect of aspirin on cancer was also investigated in clinical studies. Kune et al, found a lower incidence of colorectal cancer among subjects using aspirin-containing medication (223). In the APACC trial, patients with early stages of colorectal cancer, colorectal adenomas daily use of lysine acetylsalicylate (160 or 300mg) demonstrated a positive effect in adenoma recurrence (224). In another clinical trial, a daily low dose of aspirin (81mg) could slightly decrease the incidence of adenomas in patients compared to the placebo group after one year of treatment (225). In a randomized clinical trial, patients with familial adenomatous polyposis were treated with aspirin. Polyps were mainly detected in the epithelium of the large intestine and polyp size was decreased compared to the placebo group (226). In Lynch syndrome, which is a non-polyposis colorectal cancer type, the regular aspirin intake (600mg) also decreased the cancer incidence (227). Frouws et al. showed that regular use of aspirin (≤100mg) significantly improved patient survival with gastrointestinal cancer, including oesophageal, hepatobiliary and colorectal cancer (228), and aspirin treatment could also reduce the risk of pancreatic cancer (229). Later, Rothwell et al., studied data from five large randomized clinical trials of daily aspirin use (≥75 mg), including the United Kingdom Thrombosis Prevention Trial (TPT) (230). These studies showed that regular low-dose aspirin is associated with reduced cancer incidence of colorectal cancer both in women and men, smokers and non-smokers (230). They also demonstrated that daily aspirin intake at low dose reduces the risk of cancer metastasis, which also account for the inhibition of cancer deaths at earlier steps (231). Consistent inhibition was observed in the risk of other cancers, e.g. breast, lung and prostate cancers (230–233).

Taken together, it was a long-standing question, how aspirin treatment influences cancer progression and metastasis, whether these mechanisms are platelet-dependent and/or independent. Hypotheses supporting the role of platelet-dependent mechanisms in cancer were based not only on cellular characteristics of platelets but also pharmacodynamics of aspirin treatment and its effects on COX enzymes. The lifetime of human platelets is only 10 days, platelet turnover is very fast in the human body, therefore patients are regularly treated with aspirin every 24 hours (234, 235). 100 mg/day aspirin intake leads to maximal acetylation of circulating platelets and this could significantly reduce TxB_2_ levels, metabolite product of TxA_2_ synthesis (215, 236). Aspirin has a short half-life (approximately 20 minutes) in the blood, it is rapidly hydrolyzed to salicylic acid by enzymes located in the blood and liver (237). A low dose of aspirin intake could completely and irreversibly inhibit COX-1 activity in platelets, suggesting that aspirin uptake in platelets is a very fast process. Protein synthesis is limited in platelets compared to nucleated cells, that is due to the lack of nuclei and limited mRNA content received from megakaryocytes. Therefore the inhibitory effect of aspirin is more robust in platelets than other nucleated cells in which acetylated COXs can be easily replaced within few hours with newly synthesized enzymes (215). Although COX-1 activity is completely blocked by aspirin, acetylated COX-2 can still form 15R-hydroxyeicosatetraenoic acid (15R-HETE) from arachidonic acid (238). Furthermore, aspirin can target megakaryocytes in the bone marrow as well, therefore aspirin treatment could inhibit COX-1 function in newborn platelets, released by acetylated megakaryocytes (239). Consistently, in studies by Lucotti et al., transfusion of COX-1^+/+^ platelets resulted in increased number of B16F10 melanoma cell-induced lung metastases in thrombocytopenic mice, compared with using COX-1^-/-^ platelets (164, 240). This study summarized that aspirin has broad platelet-dependent inhibitory effects on different steps of metastasis, including (i) tumor-induced platelet aggregation, (ii) endothelial cell activation, (iii) tumor cell adhesion to the endothelium, and (iv) recruitment of monocytes/macrophages, and (v) formation of a premetastatic niche (240). Altogether, these results suggest that the anti-metastatic effects of aspirin could be mainly due to the inhibition of platelet COX-1 activity.

In an experimental cancer mouse model of human familial adenomatous polyposis, deletion of COX-2 reduces the number of polyps in the intestine, indicating a key role of COX-2 in tumorigenesis (241). Can et al. showed that aspirin intake correlates with a low risk of colorectal cancer in patients with higher expression levels of COX-2 compared to those who had lower levels or absent expression (242). Activated platelets, immune cells and also the tumor microenvironment can release different growth factors, cytokines that also stimulate gene expression of COX-2 in cancer cells. In addition, activated platelets enhance COX-2 expression in stromal cells *via* the release of IL-1β, platelet-derived growth factor (PDGF) and TGF-β, which lead to tumor progression (243). Moreover, PDGF released from platelets together with GPVI-galectin-3 interactions also triggers COX-2 production, thereby enhancing EMT in HT29 colon cancer cells (197). Altogether, these results suggest that platelets may influence tumorigenesis and cancer progression also through direct effects on COX-2, and aspirin treatment can inhibit COX-2-driven functions in a platelet-dependent manner.

COX-independent mechanisms of aspirin have also been observed. In mouse models of colorectal cancer, aspirin can induce apoptosis *via* degradation of IκBα, leading to the nuclear translocation of NF-κb (244). Inhibition of Wnt/β-catenin and extracellular signal-regulated kinase (ERK)-signaling in aspirin-treated cells was also described in different cancer cell types (245–247). However, these effects were often occured at supratherapeutic doses and concentrations of aspirin.

In summary, the majority of studies suggest a positive effect of a low dose of aspirin on cancer incidence, metastases, and cancer-associated mortality. However, long-term aspirin treatment can cause gastrointestinal bleeding complications (248), therefore alternative treatments such as TP receptor blocker or gastrointestinal-safe phosphatidylcholine (PC)-associated aspirin (249) have been proposed. The benefit and bleeding risks of aspirin-based therapy can vary depending on many factors, including age, gender, and medical history of patients [reviewed in Dovizio et al. (250)].

Additional studies are needed to confirm whether aspirin-inhibiting effects are directly associated with platelets or tumor cell or tumor microenvironment. It will be important to further study the effect of aspirin as anti-platelet medication in cancer patients with thrombotic complications. Although aspirin treatment improved adoptive T cell therapy using preclinical models of melanoma cancer (108) and prevented hepatitis B-associated hepatocellular carcinoma (251), it would be important to address whether aspirin can be used as a universal adjuvant therapy in patients with cancer.

## Purinergic Receptor P2Y_12_

The P2Y_12_ receptor is a purinergic Gi-coupled ADP receptor expressed on the platelet surface and known to regulate thrombus stability *in vivo* (252). Currently used inhibitors of P2Y_12_ are either indirectly block the receptor, such as the members of the thienopyridine family (ticlopidine, clopidogrel and prasugrel) or direct inhibitors, such as ticagrelor and cangrelor. The bioactive form of thienopyridine-derived metabolic irreversibly inhibits the binding of ADP to the receptor, leading to decreased platelet activation and aggregation responses, and reducing inside-out activation of platelet αIIbβ3 integrins (253). Clopidogrel is widely used in patients with coronary artery, cerebrovascular and peripheral vascular diseases (254). Ticagrelor and cangrelor are reversible antagonists, with similar inhibitory properties (172).

Several cancer studies in mouse models indicated the beneficial effects of these inhibitors. In orthotopic models of pancreatic cancer, clopidogrel inhibited tumor development, metastasis and the extent of cancer-associated thrombosis at a dose of 8 mg/kg, which is 4-8 fold the chronic dose in patients. This treatment probably induces complete inhibition of ADP-induced platelet aggregation (255). More recently it has been shown that in an orthotopic 4T1 breast cancer model, ticagrelor (10 mg/kg), but not clopidogrel (10 mg/kg) treatment inhibited lung metastasis and improved the survival rate in mice (256). Ticagrelor treatment was associated with reduced tumor cell-platelet aggregates in the lungs (256, 257), thereby decreasing the number of tumor metastases. In ovarian cancer models, deficiency of P2Y_12_ receptor in platelets or apyrase treatment inhibited ADP-dependent platelet-tumor cell interaction and consequent primary tumor growth (258). In platelet-mediated tumor metastasis, ADP-induced signaling enhanced the platelet AKT kinase pathway, maintained by apoptosis signal-regulating kinase 1-c-jun N-terminal kinase (ASK1-JNK)/p38-mediated phosphorylation of P2Y_12_ receptor, thereby linking ADP-induced signaling to platelet mitogen-activated protein kinase (MAPK) signaling pathways (259).

Genetic deficiency of P2Y_12_ has also been shown to inhibit lung colonization by Lewis lung carcinoma and B16F10 cells in mice. Interestingly, this effect was associated with inhibition of VEGFR1+ bone marrow-derived cell clusters, and fibronectin deposition in the lung (160). This result further supported a new anti-cancer strategy. A tumor-homing pentapeptide that targets fibrin-fibronectin complexes in the tumor stroma and the vascular wall is called CREKA, coupled to Ticagrelor (260). This CREKA-Ticagrelor complex effectively inhibited platelet-induced migration of tumor cells, and also prevented tumor-platelet interaction thereby suppressing lung metastasis (260).

In pancreatic adenocarcinoma, platelets can drive gemcitabine resistance. Platelet-released nucleotides (ADP and ATP) were found to be the main causative factor that drives gemcitabine resistance, which is completely blocked by Ticagrelor (261). Isorhapontigenin is a polyphenolic compound found in Chinese herbs and grapes with anti-cancer and anti-inflammatory properties. It can selectively inhibit ADP-induced platelet aggregation and inside-out and outside-in activation of αIIbβ3 integrins, and also δ-granule secretion. Isorhapontigenin increased adenosine 3’,5’-cyclic monophosphate (cAMP) levels and phosphorylation of vasodilator-stimulated phosphoprotein (VASP). On the other hand, decreased Akt phosphorylation was found, suggesting a potential effect of this drug on cAMP and phosphoinositide 3-kinases (PI3K) signaling pathways that are downstream effectors of P2Y_12_ receptor (262). Although clopidogrel enhanced anti-tumor and/or anti-metastatic activity of chemotherapeutic agents such as 5-fluorouracil, cyclophosphamide and mitoxantrone, on the other hand, it reduces the anti-cancer activity of doxorubicin, cisplatin and tamoxifen (263). The molecular mechanisms of such divergent activities have not yet been established, possibly based on the modulation of the tumor vasculature through platelet-released factors. It is also important to note that P2Y_12_ receptor is expressed in cells other than platelets (264), such as osteoclasts (265). Indeed, bone loss (osteolysis) associated with tumor growth in mice was effectively treated with clopidogrel (265).

Although inhibition of P2Y_12_ has beneficial effects in mouse models of cancer, the results from randomized trials are conflicting. TRITON-TIMI 38 trial of prasugrel in comparison to clopidogrel on top of aspirin during 6-15 months indicated on accelerated tumor growth and a higher risk of cancer death in the prasugrel group, in patients with breast, colorectal and prostate cancers (266). Interestingly, this effect was not observed in patients with non-melanoma type of skin cancers and brain tumors (266). In contrast, in another trial (TRILOGY), no difference has been observed in the occurrence of cancer between the clopidogrel and prasugrel group (267). In DAPT and PEGASUS-TIMI 54 trials, long-term treatment with clopidogrel and ticagrelor also showed a significant increase in cancer-related deaths (268, 269). In a retrospective study on acute coronary syndrome patients with a median follow-up of 46 months, ticagrelor treatment had lower cancer risk than clopidogrel without any difference between clopidogrel and prasugrel (270). Although these studies have been conducted in large cohorts, it is difficult to conclude any evidence of the association between the effects of P2Y_12_ inhibitors and cancer risk and related mortality. Earlier, it was hypothesized in some cases, that platelet-tumor cell trapping in the microvasculature has anti-metastatic effects, and inhibition of platelet function using these drugs may impair the barrier function of platelets (271).

### Thrombin and Thrombin Receptors

Thrombin receptors belong to a PAR family of four transmembrane GPCRs that are activated by thrombin, and trypsin-like protease-mediated cleavage of their N terminal exodomain (272, 273). PARs are expressed in platelets, neutrophils, monocytes/macrophages, endothelial cells and fibroblasts. The human platelets are mainly activated by thrombin through PAR1 and PAR4 isoforms, while mouse platelets do not express PAR1, they are activated by PAR3 and PAR4. Thrombin receptors are an attractive target for the treatment of platelet-related diseases (274). Targeting platelet PAR1 function could effectively inhibit thrombin-induced aggregation. PAR1 blocker Vorapaxar can reduce thrombotic events in patients with myocardial infarction and stroke, but moderate or severe bleeding complications were observed (275). Parmodulin targets the cytosolic part of PAR1 in reversible mode, thereby inhibiting signalings through Gαq, but not Gα12/13. Parmodulin ML-161 had anti-thrombotic and anti-inflammatory effects in mice with a lower bleeding tendency (276). Pepducin is a cell-penetrating lipidated fragment of the cytosolic part of GPCR to modulate the action of this receptor in targeted cell-signaling pathways, including different PARs. PAR1 specific Pepducin PZ-128 was proposed as an effective anti-metastatic and anti-angiogenic inhibitor in mouse models of breast, lung and ovarian cancers (277). PZ-128 has been already tested in patients with coronary diseases and resulted in decreased bleeding tendency compared to Vorapaxar (278). Interestingly, PAR1 was detected in different cancer cells, and it was cleaved by MMP1 and 13 (279). Therefore, it was proposed that targeting PAR1 function during cancer progression and metastasis would simultaneously inhibit both platelet and tumor cell functions. The clinical applicability of Parmodulin and Pepducin in cancer therapy would be important to further study in different *in vivo* experimental models of cancer.

Heparin prevents the formation of thrombin and inhibits its activity. Heparin, unfractionated heparin (UFH), low-molecular-weight heparin (LMWH) and heparin derivatives are used in the treatment of VTE and can suppress cancer cell survival (280). Heparin inhibits angiogenesis, tumor cell proliferation, adhesion, migration and invasion through the inhibition of heparanase, P and L-selectin. Furthermore, heparin treatment inhibits tumor cell-induced endothelial tube formation and CXCL12/CXCR4 signaling pathways (280). Tinzaparin is an LMWH, generated by the enzymatic degradation of porcine unfractionated heparin (UFH). Sulfated non-anti-coagulant heparin (S-NACH) is also an LMWH (281). Both LMWH effectively inhibits P-selectin-mediated cell adhesion and cancer metastasis. Modified heparin with low anti-coagulant activity decreases A375 melanoma cell adhesion to platelets through inhibition of inside-out activation of αIIbβ3 integrins (282). Heparin can also disrupt the interaction of monocyte and tumor cell-derived α4β1 with endothelial vascular cell adhesion molecule 1 (VCAM1) (283). Although the anti-cancer effects of heparin and its derivatives have been highlighted in the literature, more preclinical and clinical studies are necessary to evaluate the potential off-target effects in heparin-based platelet-cancer therapies to avoid bleeding complications.

## P Selectin

P-selectin mediates interactions of platelets with tumor cells and vasculature during tumor growth and metastasis, indicating that blockade of P-selectin exposure on the platelet surface would be a potential target for anti-cancer therapy. Rivipansel inhibits many selectins in the body, including P, L and E-selectins, and also decreases the recruitment of plasma cells to the bone marrow of multiple myeloma (284). Rivipansel treatment was tested in patients with vaso-occlusive sickle cell anemia and this treatment did not reach the necessary efficacy points (285). Recently, Crizanlizumab, a selective blocking antibody of P-selectin was tested in the same disease context with a lower incidence of adverse effects (286). Therefore, it would be important to test Crizanlizumab in different cancer mouse models in the future.

### Liquid Biopsies

Blood-based liquid biopsies are non-invasive biomarkers, which are becoming powerful diagnostic and prognostic tools to screen cancer patients (287). To evaluate tumor landscape, circulating tumor cells (CTCs) are the most commonly used liquid biopsies (287). During their transit in the bloodstream, CTCs interact with platelets and immune cells. Platelets can ingest and sequester CTC-specific proteins, mRNA and also tumor-derived pro-tumorigenic and angiogenic factors, leading to the tumor-specific modifications of platelet proteome and transcriptome. In line with this, platelets isolated from patients with glioma and prostate cancer were enriched in cancer-associated RNA biomarkers EGFRvIII and PCA (288). Best et al, detected more than 5000 differentially expressed or mutated mRNAs between healthy donors and cancer patients, including expression of MET, HER2 and mutations in KRAS, EGFR and PIK3CA (289). These datasets were effective to distinguish patients with metastatic tumors. During tumor progression, platelet transcriptome seems to dynamically change in a time-dependent manner. Therefore, it was proposed that the platelet mRNA profile allowed to accurately distinguish and predict tumor progression (290). Analyzing platelets as a reservoir of liquid biopsies would provide valuable tools for cancer diagnostics. On another hand recent studies by Dunbar et al., reported that regardless of cancer type, the mutations in genes, such as STK11, KRAS, CTNNB1, KEAP, CDKN2B and MET can predict the high incidence of cancer-associated thrombosis for one year before diagnosis (291, 292). Interestingly other genes, such as SETD2, IDH1 are not predictive and displayed a negative association (291, 292).

Soluble P-selectin and coagulation factors circulate at high levels in patients with solid cancers, predicting the cancer status and VTE complications (24, 293, 294). High plasma levels of vWF, fibrinogen and D-dimers were associated with poor prognosis of breast, colon, gastric, rectal, non-small cell lung cancer, ovarian and pancreatic cancers (295–298). Other studies observed elevated levels of TF-positive microparticles in plasma samples of patients with pancreatic, colon, breast, ovarian and non-small cell lung cancer (299). Although systematic testing of cancer patients for these pro-coagulant factors may help to identify patients at increased risk for VTE, genomic profiling of oncogenic mutations can be also useful to predict thromboembolic risks in patients with different solid cancer types. It would be important to further evaluate whether increased VTE observed in cancer patients is due to the oncogenic mutations which dysregulate the hemostatic genes in cancer cells, leading to enhanced TCIPA, thrombosis and blood clotting.

Plasma levels of soluble GPVI (sGPVI) reflect platelet activation in thrombo-inflammatory diseases, such as stroke, disseminated intravascular coagulopathy, arthritis and sepsis (300–302). GPVI can support thrombus stabilization through the interaction with fibrin and fibrinogen (200, 201, 303). Elevated levels of sGPVI in patients with sepsis were due to the GPVI shedding, which was induced by fibrin activation of this receptor (304). Recently, we found increased levels of sGPVI in plasma samples of patients with breast or colorectal cancer (142). In small cohorts of patients with colorectal cancer, sGPVI levels were positively correlated with cancer stage (142). To establish the role of sGPVI as a diagnostic/prognostic marker, future studies are needed to validate these results in a larger cohort involving other solid tumor types. Although the pro-thrombotic environment of many cancers is rich in fibrin (305), future studies are required to evaluate whether levels of sGPVI can be also a diagnostic tool to predict thrombosis and correlated to the cancer stage. GPVI would also represent a prognostic marker in pancreatic adenocarcinoma since elevated levels of GPVI were detected in microparticles isolated from patient blood (306). Further studies are required to define whether some of the above-indicated platelet-derived molecules can be universal diagnostic/prognostic markers for cancers or only specific of a particular cancer type.

## Other Alternative Targeting Strategies

It has been proposed that platelets can influence tumor growth and progression by enhancing tumor cell proliferation (307). However, the role of platelets on cancer cell proliferation remains controversial. Experimental findings were strongly dependent on the cancer cell types and *in vitro* cell culture systems. Initial studies by Ibele et al. showed that platelets have an important role in malignant tumors because leukocytes enhance the killing capacity against cancer cells in the presence of platelets (308). Another study showed that resting and thrombin-stimulated platelets exert the cytotoxic effect on K562 chronic myelogenous leukemia cells (309). Although this cytotoxic effect was abolished by esterase inhibitors in resting but not in thrombin-activated platelets (309). This phenomenon can be explained that platelets can express many immune defense factors, such as TNF, tumor necrosis factor-related apoptosis-inducing ligand (TRAIL), CD154 and Fas-L, and exposed on the platelet surface or released into the medium. The binding of Fas-L to Fas receptor (Fas-R) activates the caspase-mediated apoptotic pathway in cancer cells that express Fas-R (310).

Anoikis is a form of programmed cell death that occurs when cancer cells detached from the surrounding ECM, thereby modulating cell spreading and invasiveness (311). Interestingly, platelets induce resistance of cancer cells to anoikis (312). Platelets also enhance RhoA-MYPT1-PP1-mediated YAP1 dephosphorylation in cancer cells, thereby inducing a prosurvival gene expression signature and inhibiting apoptosis (312). Altogether, these studies suggest that platelets contain an arsenal of bioactive factors, manipulating the apoptotic cross-talks between platelets and tumor cells. In addition, platelets can induce cell proliferation of hepatocellular carcinoma by activating MAPK signaling and decreasing apoptotic mediators (313). Platelet releasate also enhances cell proliferation of human and mouse ovarian cancer, and this process is maintained by the interaction between platelet-released TGF-β and tumor-resident TGF-β receptor (314). In orthotopic xenograft, syngeneic and genetic models of ovarian and lung cancer, other platelet-derived molecules, platelet focal adhesion kinase (FAK) and PF4 enhanced platelet infiltration and tumor growth (315, 316).

Genetically modified platelets expressing TRAIL on the cell surface could eliminate cancer cells *in vitro* and significantly reduce the number of metastases in a mouse model of prostate cancer (317). Hu et al. used a platelet membrane-coated nanovehicles (PM-NV) for sequential and site-specific delivery of two anti-cancer therapeutics (TRAIL and doxorubicin) (318). PM-NV can efficiently deliver TRAIL toward cancel cell membrane to activate the extrinsic signaling pathway of apoptosis (318). Papa et al. developed detergent-extracted human-modified platelets (platelet decoys) that retained platelet binding functions but were incapable of functional activation and aggregation (319). Their results suggest that platelet decoys could represent an effective strategy for obtaining anti-metastatic and even anti-thrombotic effects (319). *In vivo* rabbit model, pretreatment with platelet decoys inhibited arterial injury-induced thromboembolism and also interfered with platelet-mediated human breast cancer cell aggregation, and decreased cancer cell arrest and extravasation in a microfluidic human microvasculature on a chip (319). In a mouse model of metastasis, simultaneous injection of the platelet decoys with tumor cells inhibited metastatic tumor growth (319).

In many studies, platelets have been proposed as a drug carrier, since platelets can easily uptake and store bioactive molecules in their secretory granules. Doxorubicin was loaded in platelets for the treatment of lymphoma. Doxorubicin-treated platelets facilitated intracellular drug accumulation through TCIPA and also could release doxorubicin into the medium in a pH-controlled manner (320). This study suggested that doxorubicin-loaded platelets could reduce the adverse effects of extracellular doxorubicin, and enhance the therapeutic efficacy in the targeted organ (320). As we mentioned above, an antibody-drug conjugate (ADC) with the combination of drug activation and release resulted in tumor cytotoxicity in a mouse xenograft model of triple-negative breast cancer (MDA-MB-231 cells) without any discernible toxic effects on other cell types (181). Loading platelets with ADC-conjugated drugs carrying to the tumor microenvironment would be a novel therapeutic approach for the treatment of a broad range of solid tumors (181).

### Platelets and Chemotherapy Resistance

Tumor chemotherapy resistance occurs when a tumor that has been responding to therapy suddenly begins to grow, and cancer cells could escape from the toxic effects of chemotherapeutic agents. This represents one of the major problems in patients with chemotherapy resistance (311) because of the higher risk to develop thromboembolic disorders than those without chemotherapy (321, 322). Clinical studies showed the correlation between platelet count and tumor chemotherapy resistance. *In vitro* studies demonstrated the contribution of increased platelet count to tumor chemotherapy resistance, when paclitaxel and 5-fluorouracil have been used for the killing of colon and ovarian cancer cells (323). In mouse models of breast and prostate carcinoma, low platelet count could increase the sensitivity to doxorubicin and paclitaxel (91, 92). Inhibition of GPVI function resulted in intratumoral hemorrhages, and this could enhance the access of the chemotherapeutic agents into the growing breast and prostate tumors (91). Platelets also contribute to the relapse of orthotopic ovarian tumors in mice after cessation of anti-angiogenic therapy with bevacizumab or pazopanib (315). Platelet FAK function is important in this process because FAK-deficient platelets completely prevented the rebound in tumor growth (315). In line with this, combined therapy with a FAK inhibitor and pazopanib/bevacizumab could inhibit the negative effects following the withdrawal of anti-angiogenic therapy (315). Altogether, it was proposed that FAK may be a unique target when anti-angiogenic agents are withdrawn, and dual inhibition of FAK and VEGF may have a therapeutic implication for ovarian cancer management (315).

Several mechanisms have been proposed that platelet releasate may also influence tumor chemotherapy resistance, thereby antagonizing the cytotoxic effect of some drugs, such as paclitaxel and 5-fluorouracil: (i) growth factors and cytokines released from activated platelets counter the anti-proliferative effects of chemotherapeutic agents by shifting the balance between anti-apoptotic and pro-apoptotic genes, (ii) platelets upregulate the regulators of cell progression thereby inducing the blocking of cell cycle arrest caused by the anti-cancer agents, (iii) platelets enhance the phosphorylation of DNA repair proteins, Chk1, BRCA1 and Mre11. Interestingly, platelets inhibited cytotoxic effects of chemotherapeutic agents sorafenib and regorafenib in hepatocellular carcinoma by increasing MAPK signaling (307). Proposed molecular mechanisms are multiple, how they can be applicable at clinical levels, warrant still many investigations.

## Conclusion

Platelets can build dynamic interactions with all other cell types in the circulation and trigger many activatory signaling pathways, thereby directly or indirectly influencing the function of tumor cells and tumor stroma. Platelets display predominantly pro-tumorigenic functions in different cancer types. Experimental and preclinical studies using knock-out animal models and a wide range of pharmacological tools provided encouraging results to consider platelets as potential targets in anti-cancer therapies. However, the clinical evidence of the beneficial effects of anti-platelet therapies in cancer is still missing. Design of optimal anti-cancer therapy in patients with active tumor malignancy is a highly challenging task. Therefore, future studies adding anti-platelet drugs into the conventional anti-cancer therapies must carefully examine many factors, including cancer type, degree of malignancy, sex, age, bleeding profile, and other risk factors, before that these drugs are applied to the patients. More experimental and preclinical studies are required to address the therapeutic value of anti-platelet strategies in solid cancers.

## Author Contributions

AB and EM-B wrote the manuscript. H-JA and TG critically reviewed the manuscript. All authors contributed to the article and approved the submitted version.

## Funding

This work was supported by the Bayersiches Landesamt für Gesundheit und Lebensmittelsicherheit (project number 15-25) and Deutsche Forschungsgemeinschaft, CRC TRR152/P15.

## Conflict of Interest

The authors declare that the research was conducted in the absence of any commercial or financial relationships that could be construed as a potential conflict of interest.
